# Headache and comorbidity in pediatric age

**DOI:** 10.1186/1824-7288-40-S1-A83

**Published:** 2014-08-11

**Authors:** Alberto Verrotti, Ilaria Bizzarri, Giulia Cecconi, Lorenza Di Genova, Manuela Vultaggio, Marta Cofini

**Affiliations:** 1 Department of Pediatrics, University of Perugia, Perugia, 06132, Italy

## Background

Frequently headache is associated with numerous comorbidities, but data are incomplete and sometimes conflicting.

The aim of this review is to highlight the relation between headache and associated disorders.

## Materials and methods

We identified studies via PubMed search indexed for MEDLINE using “*headache comorbidity and children*” as key words. The time period covered was approximately 10 years. Only English language articles were reviewed.

## Results

The findings of our research demonstrate that epilepsy, anxiety/depression, attention deficit/hyperactivity disorder (ADHD), obesity and childhood abuse are the most frequent and investigated headache-comorbidities.

Headache and epilepsy is the most frequent comorbidity in pediatric age [1-3]. The major association is with focal epilepsies, in particular criptogenetic ones [4,5]. The real effect of headache on seizure-related-headache is still unclear. Peri-ictal headache is a common feature of epileptic seizures: it can occur before, during or after seizures [6]. Although family history of headache and/or epilepsy is referred, there is not a clear association with specific type of both disorders [5].

Moreover, migraineurs have a higher prevalence of anxiety/depression than controls [7,8]. Severity of anxiety/depression is linked with severity and frequency of migraine [9]. Therefore, parents and relatives of migraneurs are affected by both headache and psychiatric disorders [10].

Another important comorbidity is headache and ADHD [11]. Headache is not associated with ADHD overall but with iperactivity/impulsivity symptoms [12]. While the relation with inactivity symptoms is controversial, differences between headache types and ADHD have been not found [13]. Structural and functional abnormalities in the brain networks seem to be central in both headache and ADHD pathophysiology [14].

It has been demonstrated an association between migraine and obesity, but the real link is still matter of debate. Psychological conditions and inflammatory mediators may be involved, as a common pathophysiologic mechanism [15]. Body mass index seems to be related to high frequency, degree of migraine attacks [16-18], and chronic migraine [19].

Many studies evidence the relationship between migraine and childhood abuse. The emotional abuses’ prevalence in migraineurs is higher than controls and it is more common in women [20]. Childhood maltreatment appears to be related to chronic and disabling headache [21]. This pattern leads to an early life stress that influences the neurobiological physiology [22].

## Conclusions

Further studies are needed to obtain detailed epidemiological data and to understand whether common mechanism(s) below these conditions exists. Physicians should consider and investigate the possible co-occurrence of these disorders in patients with headache.
